# Defect Engineering and Anisotropic Modulation of Ionic Transport in Perovskite Solid Electrolyte Li*_x_*La_(1−x)/3_NbO_3_

**DOI:** 10.3390/molecules26123559

**Published:** 2021-06-10

**Authors:** Jinhua Hong, Shunsuke Kobayashi, Akihide Kuwabara, Yumi H. Ikuhara, Yasuyuki Fujiwara, Yuichi Ikuhara

**Affiliations:** 1 Nanostructures Research Laboratory, Japan Fine Ceramics Center, Nagoya 456-8587, Japan; jinhuahong436@gmail.com (J.H.); s_kobayashi@jfcc.or.jp (S.K.); kuwabara@jfcc.or.jp (A.K.); yumi@jfcc.or.jp (Y.H.I.); 2 Faculty of Engineering, Shinshu University, Nagano 380-8553, Japan; yamato2010@shinshu-u.ac.jp; 3 Institute of Engineering Innovation, The University of Tokyo, Tokyo 113-8586, Japan

**Keywords:** defect engineering, perovskite electrolyte, lithium-ion battery, migration pathway, anisotropic response

## Abstract

Solid electrolytes, such as perovskite Li_3*x*_La_2/1−x_TiO_3_, Li*_x_*La_(1−x)/3_NbO_3_ and garnet Li_7_La_3_Zr_2_O_12_ ceramic oxides, have attracted extensive attention in lithium-ion battery research due to their good chemical stability and the improvability of their ionic conductivity with great potential in solid electrolyte battery applications. These solid oxides eliminate safety issues and cycling instability, which are common challenges in the current commercial lithium-ion batteries based on organic liquid electrolytes. However, in practical applications, structural disorders such as point defects and grain boundaries play a dominating role in the ionic transport of these solid electrolytes, where defect engineering to tailor or improve the ionic conductive property is still seldom reported. Here, we demonstrate a defect engineering approach to alter the ionic conductive channels in Li*_x_*La_(1−x)/3_NbO_3_ (*x* = 0.1~0.13) electrolytes based on the rearrangements of La sites through a quenching process. The changes in the occupancy and interstitial defects of La ions lead to anisotropic modulation of ionic conductivity with the increase in quenching temperatures. Our trial in this work on the defect engineering of quenched electrolytes will offer opportunities to optimize ionic conductivity and benefit the solid electrolyte battery applications.

## 1. Introduction

Commercial lithium-ion batteries have shaped the new era and people’s daily lives, owing to their successful portable electronics applications in vehicles and mobile phones, etc. However, there are still common issues to be addressed in this industry, such as safety, electrochemical and mechanical stability, and cycling life, which are intrinsic disadvantages of the flammable organic liquid electrolytes mostly employed in the current commercial Li-ion batteries [1,2,3,4]. Solid electrolyte batteries [5,6,7,8] or all-solid-state batteries have long been considered as the future of battery technology to avoid safety issues such as leakage and explosion, and to minimize cycling instability due to side reactions or metal dendrite growth. Thus, solid electrolyte materials [9,10,11,12] have attracted extensive research interest in the advanced architecture design [13,14] of Li-ion batteries and Li-air, Li-S [15], and Li-Br_2_ batteries [16,17] with exceptionally high energy density.

Among the available lithium-ion-conducting solid electrolytes, ceramic oxides (10^−5^~10^−3^ S·cm^−1^) such as perovskite materials Li_3*x*_La_2/1−x_TiO_3_ (LLTO) [18,19,20,21] and Li*_x_*La_(1−x)/3_NbO_3_ (LLNO) [22,23,24], anti-perovskite Li_3_OX (X = Cl, Br)[25,26,27] and garnet structured Li_7_La_3_Zr_2_O_12_ (LLZO) [28,29,30,31] have received much attention due to their good electrochemical stability and considerable potential to push the limit of ionic conductivity towards a desired level (~10^−2^ S·cm^−1^) in the industrial application of batteries. Their ionic conductivity follows such a microscopic ion migration mechanism: low occupancy of the Li^+^ on the vacancy sites; low migration energy barrier for ion hopping; and network-like available sites (vacancies) [32] to interconnect the migration pathways of the mobile ions in these solid electrolytes [1]. In practical battery applications, the presence of point defects, grain/domain boundaries [21,33] in polycrystalline materials, and electrolyte/electrode interface resistance [34,35] would play a dominant role in determining the Li ionic conductive performance.

Ma et al. [21,33] have demonstrated the atomic structure of grain boundaries in polycrystalline oxide electrolytes which severely degrade the ionic conductivity compared to the perfect bulk form. Very recently, Dawson et al. [36] utilized large-scale molecular dynamic simulations to show that the grain size distribution of the polycrystalline electrolytes is key to the overall ionic transport. The conductivity has a strong decrease [36] when the grain size is smaller than 100 nm, and it increases and converges to 85% of the conductivity of perfect bulk crystal when grain size exceeds 400 nm. The directional solidification method has been demonstrated to yield high-quality single-crystal electrolyte ingots at the decimetre scale [37], where the limited grain boundaries (if they exist between large-size domains) may not remarkably influence the macroscopic ionic conductivity. Herein, atomic structure characterization of other common structural disorders is also quite necessary to understand their effect on the ion diffusion. Furthermore, there have not been adequate experimental reports on how to utilize defect engineering to perfect or tailor the ionic conductivity at the atomic scale.

In this work, we will show the formation of various defects by the rearrangement of La sites in Li*_x_*La_(1−x)/3_NbO_3_ (LLNO, *x* = 0.1~0.13) electrolytes after a quenching process and that the resulted La interstitials/vacancies induce the anisotropic change in the ionic conductivity. Through aberration-corrected scanning transmission electron microscopy (STEM), we have identified the layered structure property of LLNO by atomic resolution annular dark/bright field (ADF/ABF) imaging, together with energy dispersive X-ray spectroscopy (EDX) mapping to visualize the layered chemical structure in atomic resolution. The experimental quenching process of single-crystal LLNO gives rise to vast La vacancy defects in the La, Li-coexisting A_1_ layer and octahedral interstitial La atoms occupied in the “empty” O-containing A_2_ layer. This La rearrangement mechanism leads to the anisotropic experimental findings that the in-A_1_-plane conductivity increased, while the out-of-A_1_-plane conductivity decreased when the quenching temperature elevated. Based on this microscopic diagram, the migration of La atoms along different kinetic pathways results in the formation of in-A_1_-plane vacancies and in-A_2_-plane interstitials which are responsible for the anisotropic modulation of ionic conductivity through defect engineering. Our trial in this work on the defect characterization of the quenched single-crystal LLNO will offer new opportunities to optimize the ionic conductivity and benefit in its potential applications in the new solid electrolyte batteries.

## 2. Results and Discussion

**Figure 1:**
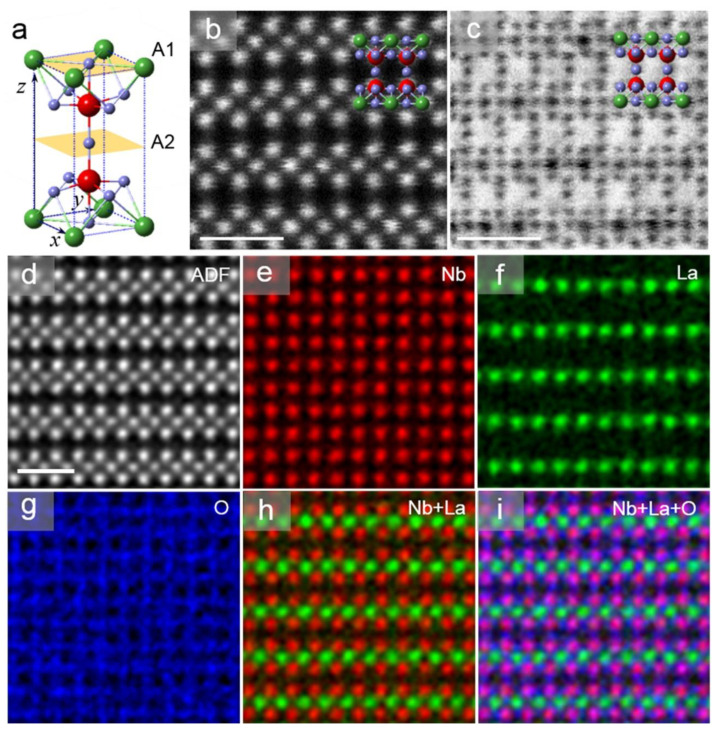
(**a**) Structure model of perovskite electrolyte LLNO. In the unit cell, green atoms stand for coexisting Li, La sites with an occupancy of 2/3, red for Nb with 100% occupancy, and blue for O with 100% occupancy. The A_1_ layer marks the La, Li-coexisting layer, and A_2_ layer is the “empty” mid-plane gap between A_1_ layers. (**b**,**c**) Atomically resolved HAADF and ABF images in [100] direction, respectively. The atomic models superposed suggest the invisible O atoms can be clearly identified in the ABF image. (**d**–**i**) STEM-EDX mappings to reveal the chemical structure. Atomic resolution EDX mapping depicts the layered distribution of La in A_1_ plane which is partly replaced by Li ions. Scale bars: 1 nm.

Solid electrolyte Li*_x_*La_(1−x)/3_NbO_3_ (LLNO) has a perovskite structure, as shown in Figure 1a, where the La atoms (green balls) are located at the vertex sites with 2/3 occupancy. This means 1/3 of the vertex La sites are inherent vacancies which accommodate Li ions. Hence, this special Li-containing plane is named as the A_1_ layer [23,24] with rich vacancy networks, and the middle-plane gap between neighbouring A_1_ layers is called the A_2_ layer, which contains only O atoms (purple in Figure 1a). In the charging or discharging process of the Li ion battery, these layers could be the Li^+^-conductive channels for ionic transport in the solid electrolyte.

Figure 1b,c show the atomic resolution high angle annular dark/bright field scanning transmission electron microscopy (HAADF/ABF-STEM) images of the Li*_x_*La_(1−x)/3_NbO_3_ (*x ≈* 0.13) crystal in the [100] direction. Heavy atoms La and Nb are clearly presented in the HAADF image, while the O columns invisible in HAADF imaging can be unambiguously resolved in the ABF image in Figure 1c. These atomically resolved images demonstrate that there exist no interstitial La atoms in the A_2_ layer of the pristine electrolyte crystal.

Furthermore, energy dispersive X-ray spectroscopy (EDX) mappings clearly show the atom-by-atom chemical identification of LLNO with distinguished layered or squared distribution of La, Nb and O lattices, as shown in Figure 1d–i. These EDX mappings show that there exist only La and O in the A_1_ plane, and the layered La distribution (green in Figure 1f) also indicates the ionic conductive channel, since Li replaces part of the La atoms within the A_1_ plane in the pristine LLNO. As a supplement to EDX detection, electron energy loss spectroscopy (EELS) demonstrated a weak signal around 60 eV that originated from the Li-K edge as shown in Figure S1, which indicates the low content of Li in the pristine samples.

In order to improve the ionic conductivity properties of the single-crystal electrolyte, material processes such as annealing and quenching were conducted to tailor the microstructures of the materials [22]. These high-temperature material processes result in ordered structure modulation [23], atom migration, new defects’ formation, phase segregation or precipitation. Hence, defect engineering by materials annealing/quenching is to be explored to discover new atomic mechanisms for the enhancement of the ionic conductivity.

**Figure 2:**
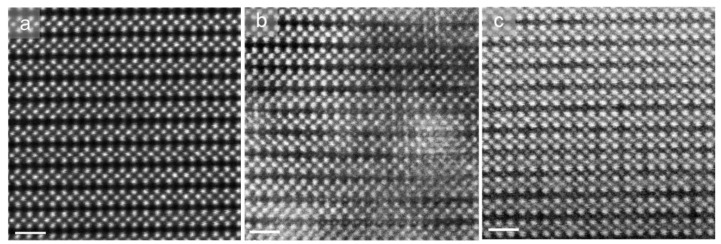
(**a**–**c**) Structure evolution of pristine and 700 °C-, 1000 °C-, and 1300 °C-quenched LLNO electrolytes, respectively, imaged along [100] direction. Scale bars: 1 nm. Defects appear with the increase in quenching temperatures. The “empty” O-containing A_2_ layers in the pristine and 700 °C-quenched electrolytes in (**a**) underwent a complex transition with diverse defects precipitating in (**b**), and became fully occupied by interstitials in (**c**) at the high temperature of 1300 °C.

Figure 2a–c show the HAADF images of the electrolytes quenched at different temperatures. In contrast with the pristine and 700 °C quenched samples (almost the same) in Figure 2a, more defects such as interstitial atoms and novel complicated intermediate precipitates emerge in the samples quenched at the higher temperature of 1000 °C (Figure 2b). For crystals quenched at the even higher temperature of 1300 °C in Figure 2c, the interstitial atoms fully occupy the “empty” A_2_ layer which should contain only HAADF-invisible O atoms. One can also see the more obvious differences in the HAADF images in Figures S2 and S3.

**Figure 3:**
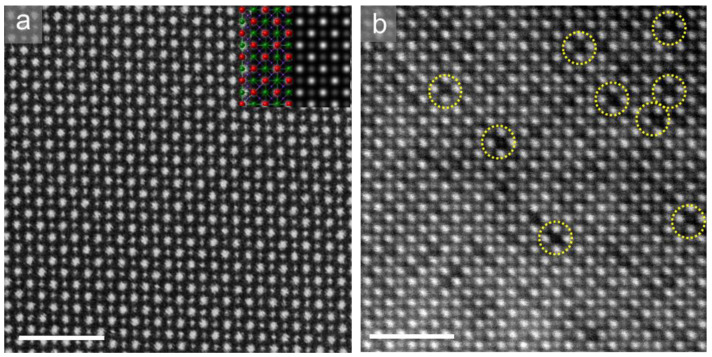
Atomically resolved HAADF images of pristine (**a**) and 1300 °C-quenched (**b**) LLNO electrolytes along [001] direction. Scale bars: 2 nm. La vacancies are not present in pristine non-quenched samples, where the inset simulated HAADF image in (**a**) agrees well with the experimental image. Dark atomic columns correspond to La in the inset of (**a**), as indicated by the green atoms (La) in the superposed structure model. Vast La vacancies appear in the 1300 °C-quenched sample, as highlighted by the yellow circles in (**b**).

As shown in the HAADF images in the [001] direction in Figure 3, in contrast with the uniform square lattice of the pristine non-quenched electrolytes, vast vacancies appear in the 1300 °C quenched samples, highlighted by yellow circles. The simulated HAADF image in the Figure 3a inset shows the dark atoms correspond to the green La atoms in the superposed structural model. This confirms that vast La atoms move out of the original A1 layer and leave vacancies in the samples when quenched at high temperatures. Therefore, a rearrangement of La lattice atoms has occurred where La atoms within the A_1_ plane migrate into the “empty” A_2_ layer, behaving as the obvious “interstitial” defects in Figure 2c. From the serial images Figure 2a–c, it is also reasonable to propose that the regular “interstitial” defects (in Figure 2c) could be a metastable configuration in the La migration.

**Figure 4:**
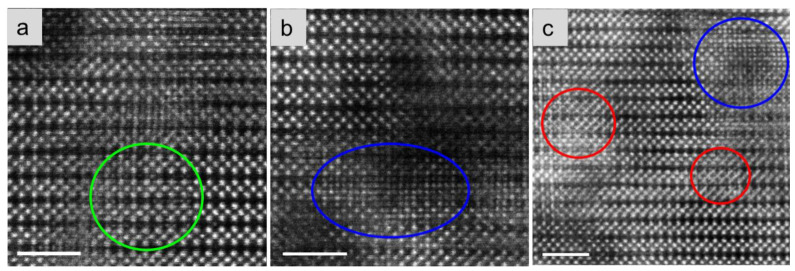
(**a**–**c**) The diverse defects formed in the 1000 °C-quenched electrolytes containing many intermediate defects, highlighted by ellipses in different colours. Scale bars: 2 nm.

However, for the electrolytes quenched at the intermediate temperature 1000 °C (Figure 2b), much more diverse “precipitated” defects with unique atomic structures appear, shown in Figure 4a–c and also highlighted by ellipses in different colours. After analysing the diverse defects formed in the 1000 °C-quenched samples, only three types of defects are found to be the most common, highlighted by ellipses in colours standing for specific atomic structures. Initial interpretation of the emerging of diverse defects in the annealing/quenching process will also be discussed later.

**Figure 5:**
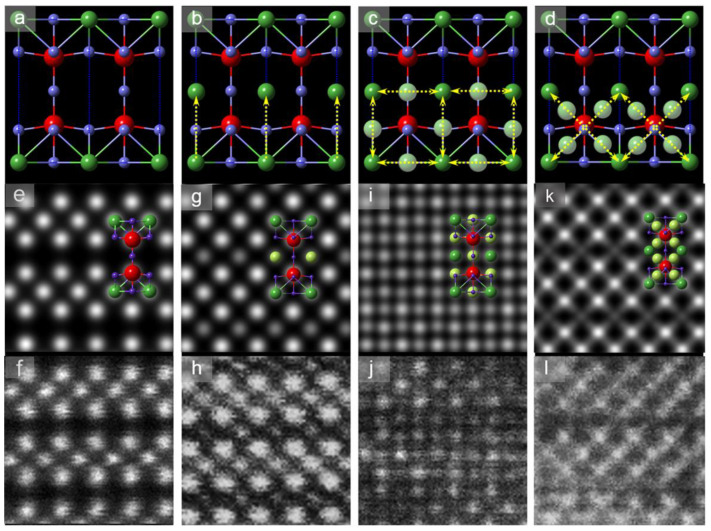
(**a**,**b**) Structure models of the ground state and metastable states. Note that the green La atoms have a probability of 1/3 to be a vacancy even in the pristine electrolyte. La_A1_→La_A2_ migration leads to the metastable state in (**b**), where yellow arrows mark the kinetic pathways. Consistent with the previous models, red balls stand for Nb, green for La/Li and purple for O. (**c**,**d**) Nearest-neighbouring and second-nearest-neighbouring La-site migration, together with interstitially trapped La (light green within the yellow arrows). (**e**,**f**) Simulated and experimental HAADF images of ground-state pristine lattice. (**g**,**h**) Simulated and experimental HAADF images of metastable defects in (**b**). (**i**–**l**) Simulated and experimental HAADF images of diverse defects corresponding to different La migration pathways. Structure models are overlaid on the simulated images in (**e**,**g**,**i**,**k**), which are consistent with the experimental images.

To better illustrate the atomic structure, we present the models and experimental HAADF images of different defects systematically in Figure 5b–d,h,j,l. After careful comparison of these defects with the normal layered matrix lattice in Figure 5a,e,f, it can be inferred that the diverse occupancies of interstitial sites must stem from La atomic migration via different kinetic pathways. As the “empty” A_2_ layer has adequate space to accommodate migrating La atoms activated by high temperature annealing/quenching, the La_A1_→La_A2_ migration will easily occur along the proposed kinetic pathway, as marked by the yellow arrows in Figure 5b. This will lead to the most commonly observed metastable defects in Figure 2c. On the other hand, as depicted previously, 2/3 of La sites within the A_1_ layer are occupied by La atoms and the left 1/3 La sites correspond to vacancies or Li in the pristine electrolyte LLNO. Hence, the nearest-neighbouring La-site hopping (indicated by arrows in Figure 5c) or second-nearest-neighbouring La-site hopping (arrows in Figure 5d) will take place via the vacancy mechanism.

Following these migration pathway hypotheses, the normal La_A1_ in Figure 5a can be regarded as the ground state configuration and La_A2_ in Figure 5b as the metastable state. Then, vacancies in these ground/metastable configurations offer considerable possibilities to the transitions between both states, leaving diverse intermediate defects (light green in Figure 5c,d) in the nearest- and second-nearest-neighbouring hopping pathways. Based on the structural models in Figure 5a,b, the simulated HAADF images of the ground-state/metastable configuration (Figure 5e,g, respectively) are consistent with the experimental results of pristine and quenched electrolytes in Figure 5f,h and Figure 2a,c. One can also see the effect of different La_A2_ occupancies on the quantitative intensity of the simulated HAADF image in Figure S4. Other diverse configurations trapped by quenching at the intermediate temperature in Figure 5c,d will contribute to abnormal lattice imaging, as simulated in Figure 5i,k, respectively, in accordance with the experimental observations in Figure 5j,l. Note that only a part of the La_A1_ and La_A2_ sites preserves vacancies, hence intermediate defects due to random migration of La should be superposed onto the perfect lattice without La vacancies in the image simulation, leading to a better matching between Figure 5i,k and Figure 5j,l. The qualitative experiment/simulation agreement confirms the diverse La-migration-induced defects between normal lattices.

To interpret the formation of these defects, we propose a temperature-dependent configuration diagram. The pristine unit cell configuration has the lowest formation energy, with all La situated in a close-packing polyhedron formed by 12 neighbouring O atoms (Figure 1a), corresponding to the ground state. High-temperature thermal activation may break the La-O bonds and yield the in-A_1_-plane, in-A_2_-plane, and inter-plane La_A1_→La_A2_ migrations when there exist plentiful vacancies or empty A_2_ sites to accommodate La. A finite-time thermal process allows the mobile La to reach metastable sites with a lower coordination number (Figure 2c, Figure 4 and Figure 5) compared with the pristine La sites, yielding metastable phases with higher formation energies. The complicated kinetic pathways (Figure 5) for atomic migration in solids contribute to different metastable configurations after the finite-time thermal process, resulting in the formation of diverse defects. This resembles the Martensitic transformation of quenched steel in a diffusionless and military manner.

**Figure 6:**
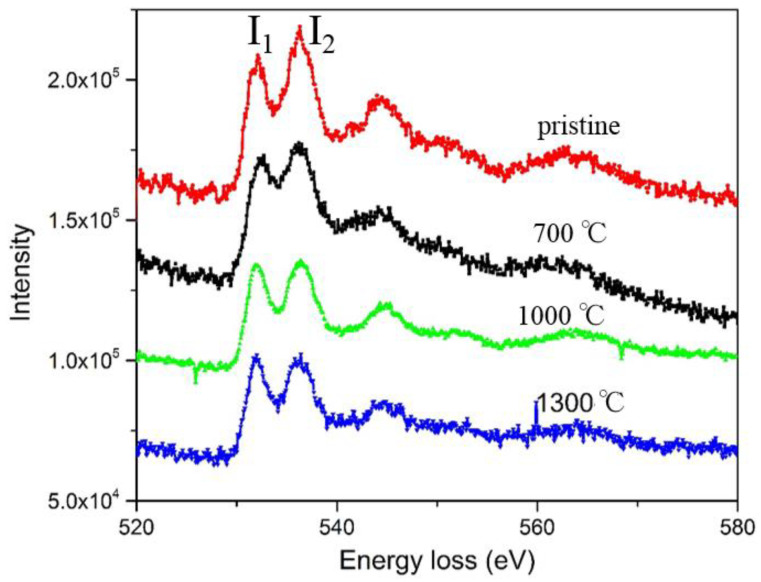
The O K-edges of differently quenched single-crystal electrolytes. The relative ratio of double peaks I_1_/I_2_ changes obviously with the quenching temperature, highly correlated with the metastable La_A2_ formation.

The La_A1_→La_A2_ transition not only generates the emerging of the metastable configurations, but also creates a new chemical environment and coordination structure of the O ligands which can be detected by energy loss spectrum near-edge fine structures (ELNES). As shown in Figure 6, the K edges of O atoms (1s→final state) with double peaks, denoted as I_1_ and I_2_, demonstrate obvious variations in differently quenched samples. The double peaks in the O K-edge are mainly caused by t_2g_ and e_g_ splitting due to hybridization of unoccupied O 2*p* orbitals with Nb 3*d*^0^ orbitals [38] in the crystal field of octahedral O-ligands surrounding Nb. From the atomic structure characterization in Figure 2 and Figure 4, the samples quenched at 1000 °C contain both perfect lattice and diverse precipitated defects, acting as an intermediate mixture of the pristine and 1300 °C-quenched samples. Although no obvious chemical shift occurs at the O K-edges, the intensity ratio of double peaks (I_1_/I_2_) increases with the quenching temperature. This indicates us that the metastable La_A2_ configuration contributes to the high I_1_/I_2_ ratio. As most O atoms reside outside of the A_2_ planes and are less coordinated with La after the La_A1_→La_A2_ transition, this indicates a higher unoccupied density of states of the hybridized e_g_ orbitals. Besides the ELNES of O, the M_2,3_ edges of Nb also present variations when the quenching temperature increases (Figure S5), resulting from atomic migration and coordination rearrangement.

**Figure 7:**
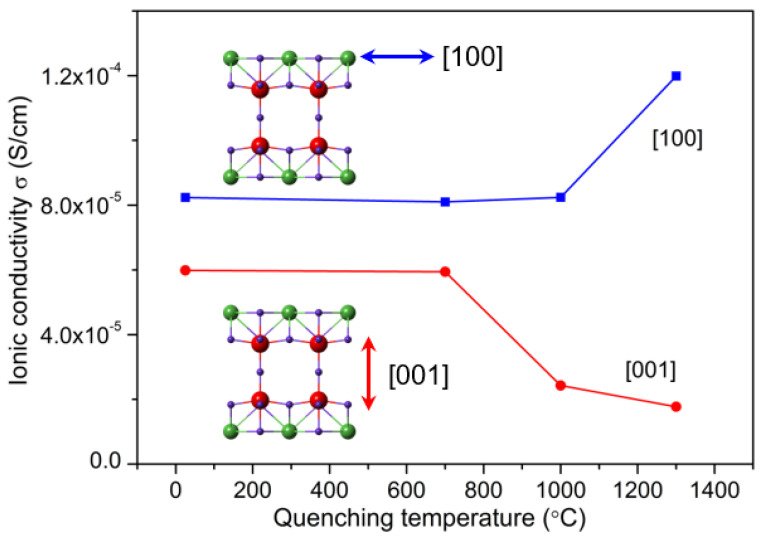
The anisotropic change of the ionic conductivity with the increasing quenching temperature. The in-A_1_-plane conductivity (in [100] direction) increases due to the La_A1_→La_A2_ transition, forming more vacancies and space for ionic diffusion. As a result, the presence of La_A2_ and other intermediate defects block the out-of-A_1_-plane pathway (in [001] direction).

In the LLNO electrolytes, vast La vacancies within the A_1_ layer emerge and the La_A1_→La_A2_ transition occurs under high-temperature annealing/quenching. This will give rise to the enhancement of the in-A_1_-plane ionic conductivity, since part of La_A1_ moves out of the Li migration pathways. In contrast, the out-of-A_1_-plane (perpendicular to A_1_ plane) Li ion transport would be partly blocked due to the presence of La_A2_ interstitial defects (Figure 2c). As seen in Figure 7, the conductivity in the [100] direction (in-A_1_-plane) increases by almost one order of magnitude to 10^−4^ S·cm^−1^, while the conductivity in the [001] direction (out-of-A_1_-plane) decreases by three times as the quenching temperature increases. Note the measurements are all conducted on quenched single-crystal electrolytes. As a solid electrolyte, LLNO is an electronic insulator, and its macroscopic ionic conductivity is dominated by mobile ions with small radii such as Li^+^. In our EELS measurement, we did not detect any local reduction of the valence state of Nb^5+^ in the single-crystal LLNO, which avoided the appearance of electronic conductivity. Local minor oxygen vacancies may exist and compensate the small amount of Li deficiency induced by the heat treatment. It is well known that oxide-ion conductivity usually appears at elevated temperatures [39]. We suppose that oxide ion conductions in the quenched LLNO samples hardly contribute to the total conductivities at room temperature.

**Figure 8:**
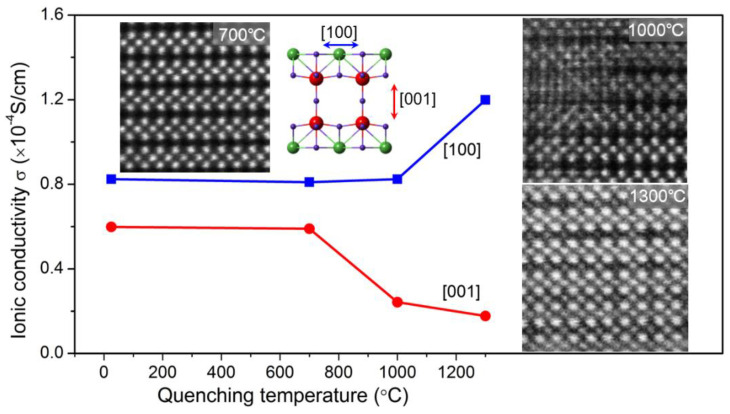
Quenching-induced defects and anisotropic changes in ionic conductivity.

Defect engineering through atomic migration in the high-temperature quenching accounts for the anisotropic modulation of Li ionic transport (Figure 8). This can be interpreted by the anisotropic Li^+^-migration energy barriers with the presence of La-vacancies in the A_1_ layer and metastable La_A2_ interstitials in the “empty” A_2_ layer. The former La vacancies will obviously decrease the energy barrier of the Li^+^ in-A_1_-plane migration due to the extra space freedom for Li ionic diffusion, and the latter interstitial La_A2_ atoms will increase the energy barrier of the Li^+^ out-of-A_1_-plane migration. Anisotropic modulation of ionic conductivity through this defect formation mechanism will also offer a possibility to tune the ionic conductive pathways in the future design of solid electrolytes in battery applications.

## 3. Sample Preparation and Characterization

Single-phased Li*_x_*La_(1−x)/3_NbO_3_ sintered bodies were synthesized according to the following formula, where the desired Li composition was determined.

*x*LiNbO_3_ + (1−x)La_1/3_NbO_3_ → Li*_x_*La_(1−x)/3_NbO_3_

The Li*_x_*La_(1−x)/3_NbO_3_ sintered body placed in the platinum crucible was melted at 1362 °C, then solidified to grow the single crystal by crucible translation in the vertical temperature gradient furnace [37]. To maintain structural stability, the Li content should be kept as low as *x* < 0.2 to obtain high-quality single crystals. The prepared Li*_x_*La_(1−x)/3_NbO_3_ single crystals were annealed at 700 °C, 1000 °C and 1300 °C for ten minutes and then quenched to room temperature. The Li contents of the quenched samples were hardly changed in comparison to that of the pristine single crystal measured by inductive couple plasma (ICP) spectroscopy. The wafers for ionic conductivity measurement and microstructure observation were prepared by cutting the LLNO single crystals.

The ionic conductivity was measured by impedance spectroscopy in a 2-electrode setup using a Hewlett Packard 4192A impedance analyser, operated at 50 Hz~13 MHz, in air and at room temperature. The (001) and (100) surfaces of the samples were polished and then sputter-coated by Au to serve as the electrodes.

Through grinding the single-crystal wafer in ethanol, thin flakes of electrolytes were deposited onto a Cu TEM grid with holey carbon film for STEM observation. Samples were cleaned in a plasma cleaner (JIC-410, JEOL Ltd., Tokyo, Japan) to remove surface contamination or the amorphous layer before the TEM characterization. Atomically resolved HAADF and ABF imaging were conducted on an aberration-corrected TEM (JEM-2100F, JEOL Ltd., Tokyo, Japan) at 200 kV. The convergence angle of the incident electron probe was set to 25 mrad, the HAADF acceptance angle was 70–240 mrad, and the ABF detector 11–22 mrad. EEL spectra were obtained using an EEL spectrometer (Tridiem ERS, Gatan, Inc., Warrendale, PA, USA) attached to a Wien filter monochromated aberration-corrected STEM (JEM-2400FCS, JEOL Ltd., Tokyo, Japan) operated at 200 kV. EEL spectra were recorded in STEM mode, using 0.1 eV per channel and an energy resolution of 300 meV (full-width at half-maximum of zero-loss peak). The convergence and collection semi-angles were 33 and 43 mrad, respectively. Atomic resolution STEM-EDX mapping was performed on a JEM-ARM200CF (JEOL Ltd., Tokyo, Japan) at 200 kV, equipped with a double large-window silicon drift EDX detector forming a large effective collection solid angle of 1.7 Sr. HAADF image simulation was performed by the software QSTEM (C.T. Koch, Berlin, Germany) to match the experimental results.

## 4. Conclusions

In summary, the diverse defects emerging in the quenching process of LLNO were systematically characterized by atomically resolved HAADF-STEM imaging and were revealed as the ground/metastable configurations involved in different kinetic pathways of La atoms. This further suggests the diverse atomic processes and energy barriers of La migration via vacancy mechanisms. Meanwhile, the La vacancies’ rearrangement in the La_A1_→La_A2_ transition leads to the anisotropic response of the ionic conductivity to the increasing quenching temperature of the electrolytes. Our investigation on this perovskite electrolyte has provided the possibility of tuning ionic conductivity through specific defect engineering to promote its application in solid electrolyte batteries or all-solid-state batteries.

## Data Availability

The data presented in this study are available on request from the corresponding author.

## Supplementary Materials

Supporting information is available online, Figures S1 and S5: Core level EELS of solid electrolytes LLNO. Figures S2 and S3: Vacancies or interstitial defects in the quenched electrolytes LLNO. Figure S4: Simulated HAADF image of LLNO in [100] direction with different A_2_-layer occupancies.

## Sample Availability

The samples in this study are available for only research purpose on request from the corresponding author.
