# Supplementary material for: Defect Engineering and Anisotropic Modulation of Ionic Transport in Perovskite Solid Electrolyte LixLa(1−x)/3NbO3
Source: Molecules. 2021 Jun 10;26(12):3559. doi: 10.3390/molecules26123559 (PMC8230448; doi:10.3390/molecules26123559)
Supplement: Supplementary file 1 [file molecules-26-03559-s001.zip › molecules-1229573-supplementary.pdf]

Supporting Materials

Defect Engineering and Anisotropic Modulation of Ionic Transport in Perovskite Solid Electrolyte $Li_xLa_{(1-x)/3}NbO_3$

Jinhua Hong [1], Shunsuke Kobayashi [1], Akihide Kuwabara [1], Yumi H. Ikuhara [1], Yasuyuki Fujiwara [2] and Yuichi Ikuhara [1,3,*]

[1] Nanostructures Research Laboratory, Japan Fine Ceramics Center, Nagoya 456-8587, Japan, jinhuahong436@gmail.com (J.H.); s_kobayashi@jfcc.or.jp (S.K.); kuwabara@jfcc.or.jp (A.K.); yumi@jfcc.or.jp (Y.H.I.)
[2] Faculty of Engineering, Shinshu University, Nagano 380-8553, Japan, yamato2010@shinshu-u.ac.jp
[3] Institute of Engineering Innovation, The University of Tokyo, Tokyo 113-8586, Japan
* Correspondence: ikuhara@sigma.t.u-tokyo.ac.jp

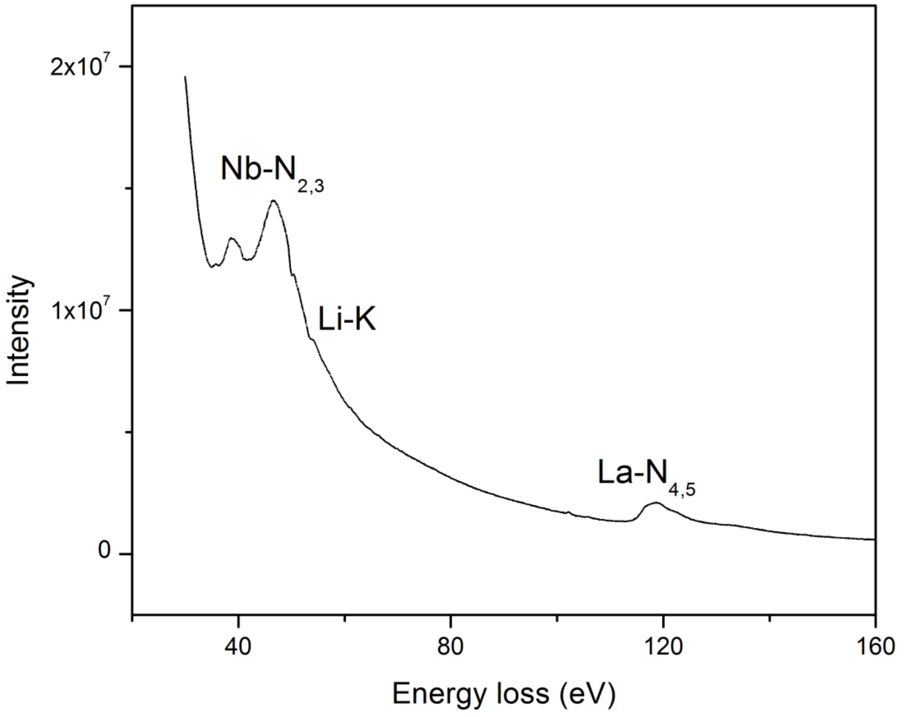

Figure S1. Core-level EELS of solid electrolyte LLNO to show the low content of Li.

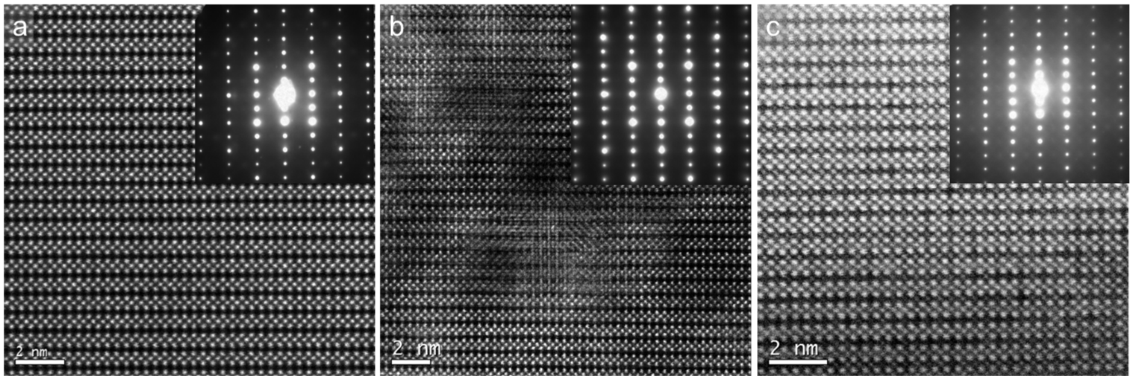

Figure S2. Atomically-resolved HAADF images of electrolytes quenched at different temperatures. (a) pristine non-quenched and 700℃-quenched. (b) 1000℃-quenched. (c) 1300℃-quenched. The corresponding diffraction patterns show no obvious changes except the spot intensity, while the dark patterned spot arrays in 1300℃-quenched indicate the emerging of a ordered metastable structure.

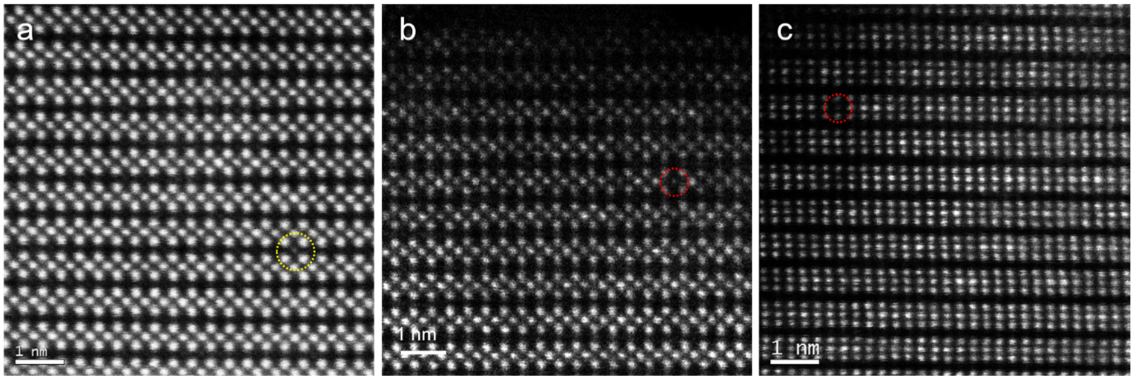

Figure S3. Atomically-resolved HAADF images of 700℃-quenched electrolytes along [100] direction (a,b) and [110] direction (c). Yellow and red circles indicate La interstitials and vacancies, respectively.

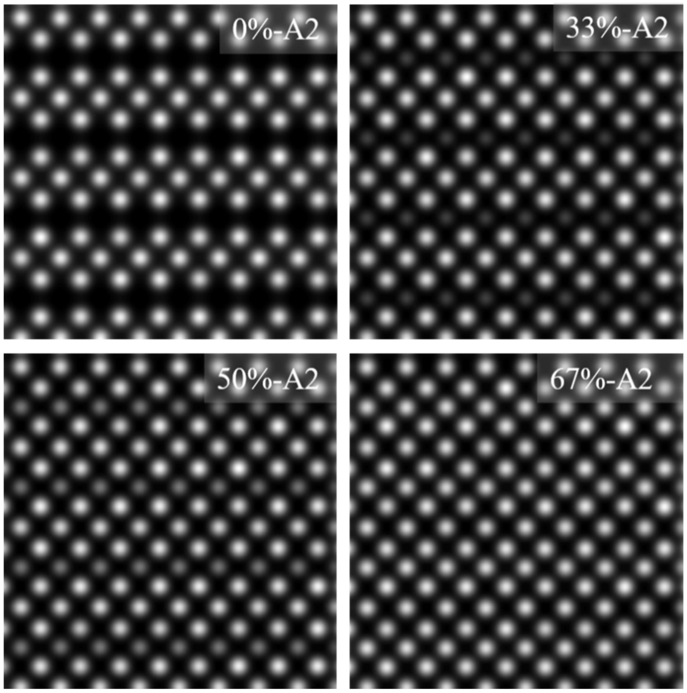

Figure S4. Simulated HAADF image of LLNO electrolytes along [100] direction with different La_{A2} occupancies. Note that 0% occupancy in A2 layer corresponds to the perfect lattice, and the La occupancy in A1 layer in the perfect crystal is 67% and the left 33% are vacancy sites to accommodate Li ions. All these simulations are based on the structures in which La_{A1} occupancy keep as a constant (67%) and La_{A2} occupancy changes.

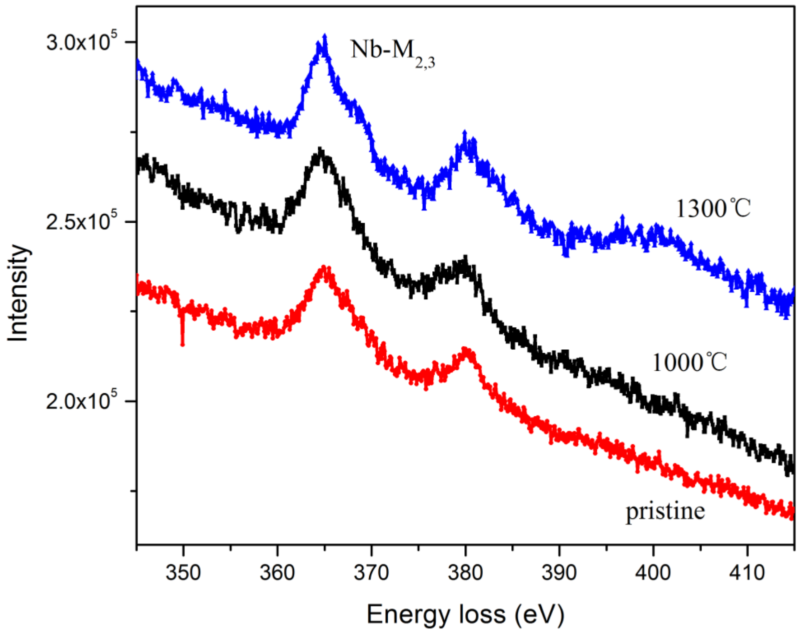

Figure S5. EELS-ELNES of Nb in solid electrolyte LLNO quenched at different temperatures. The intensities of Nb-M_2 at 375eV and Nb-M_1 at 400eV increase with the quenching temperature, resulted from the more coordinated Nb after the atom rearrangement at high temperature.
